# Relationship between electrophysiological performance during an incidental memory task and cholinergic integrity in a pilot study in adults with Down syndrome

**DOI:** 10.1002/alz.095622

**Published:** 2025-01-09

**Authors:** Alexander C Conley, Jason K Russell, John Patrick Begnoche, Brian Boyd, Alexandra P Key, Adam J Rosenberg, Allison Stranick, Rachel Schlossberg, Dann Martin, Lealani Mae Y. Acosta, Jo Ellen Wilson, Michael Rafii, Paul A Newhouse

**Affiliations:** ^1^ Center for Cognitive Medicine, Vanderbilt University Medical Center, Nashville, TN USA; ^2^ Vanderbilt University Medical Center, Nashville, TN USA; ^3^ Department of Pediatrics, Emory University Medical Center, Atlanta, GA USA; ^4^ Alzheimer’s Therapeutic Research Institute, University of Southern California, San Diego, CA USA; ^5^ GRECC, VA, Tennessee Valley Healthcare System, Nashville, TN USA

## Abstract

**Background:**

Adults with Down syndrome (DS) are at a high risk of developing Alzheimer’s disease (AD) due to the triplication of the amyloid precursor protein on chromosome 21. Despite the high incidence of AD within the DS population, there is less understanding of how AD progresses, although it may be reflected in an accelerated aging phenotype. Compared to typically developing populations, there is less understanding of the decline of cholinergic integrity with aging in adults with DS. In this pilot study we are investigating the impact of cholinergic system integrity using [^18^F]‐FEOBV PET on cortical structure and cognitive function in adults with DS. One measure of cognitive function that we used was attention and memory processes recorded by high‐density electroencephalography (EEG).

**Method:**

Nine non‐demented adults with DS (4 female, aged 37.8±7.7 years), recruited from the Trial Ready Cohort – Down syndrome, completed an MRI, [^18^F]‐FEOBV PET scan and an EEG recording. Participants completed an incidental memory task, a passive memory task with a series of complex images half of which are novel, and half repeated. Oscillatory power across the frontal, central and parietal regions was defined as a repeated–novel power. [^18^F]‐FEOBV PET images were coregistered to structural MR images and referenced to the supraventricular white matter. Initial analyses focused on memory‐related evoked power from the delta, theta, alpha and beta frequency bands, and their association with [18F]‐FEOBV PET uptake.

**Result:**

Unexpectedly, recognition memory for repeat vs novel stimuli was associated with reduced power for delta, theta and beta bands and increased alpha power. This pattern of results reflect memory processes identified with older adults in the typically developing population rather than an age‐matched sample. Increased cholinergic uptake was associated with this pattern of older age‐like power across all frequency bands.

**Conclusion:**

Evoked oscillatory activity on a recognition memory task in a small sample of adults with DS displayed similar characteristics as older adults in the typically developing population. Cholinergic integrity was associated with evoked power during recognition memory. These results demonstrate functional markers of accelerated aging in adults with DS as well as their relationship with cholinergic integrity.

